# Supplementary material for: Cattle management in an Iron Age/Roman settlement in the Netherlands: Archaeozoological and stable isotope analysis
Source: PLoS One. 2021 Oct 1;16(10):e0258234. doi: 10.1371/journal.pone.0258234 (PMC8486104; doi:10.1371/journal.pone.0258234)
Supplement: S1 Table — (PDF) [file pone.0258234.s001.pdf]

S1 Table. Context information on the specimens used in this study.
All specimens are from the archaeological site of Houten-Castellum, province of Gelderland, the Netherlands (toponym: HTN-CAS-10, coordinates 52.0173, 5.1809).
Specimens are currently held at the Institut für Prähistorische Archäologie, Freie Universität, Fabeckstraße 23-25, 14195, Berlin, Germany.
No permits were required for the described study, which complied with all relevant regulations.

**Lab nr**	**Trench**	**Feature**	**Find nr**	**Species**	**Element**	**Left/right**	**Period**	**Date**
MG08	9	67	147	*Bos taurus*	M3i	L	MR A	AD 70-120
MG04	14	43	129	*Bos taurus*	M3i	R	LIA A	250-120 BC
MG06	14	25	135	*Bos taurus*	M3i	R	MIA B	400/375-250 BC
MG24	15	3	70	*Bos taurus*	M3i	R	MR A	AD 70-120
MG13	22	101	378	*Bos taurus*	M3i	L	LIA A	250-120 BC
MG31	29	12	27	*Bos taurus*	M3i	R	MIA B	400/375-250 BC
MG33	29	29	53	*Bos taurus*	M3i	L	LIA A	250-120 BC
MG38	29	33	90	*Bos taurus*	M3i	R	ER B	AD 40-70
MG40	36	89	345	*Bos taurus*	M3i	L	MIA B	400/375-250 BC
MG26	41	20	136	*Bos taurus*	M3i	R	MR A	AD 70-120
