# Supplementary material for: Cattle management in an Iron Age/Roman settlement in the Netherlands: Archaeozoological and stable isotope analysis
Source: PLoS One. 2021 Oct 1;16(10):e0258234. doi: 10.1371/journal.pone.0258234 (PMC8486104; doi:10.1371/journal.pone.0258234)
Supplement: S2 Table — Wear stages after Grant (1982) [23]. (PDF) [file pone.0258234.s002.pdf]

S2 Table. Intra-tooth oxygen and carbon isotope ratios of enamel from cattle teeth from Houten-Castellum. Wear stages after Grant (1982).

**MG04, Late Iron Age A, M3i R, wear stage g, cusp to ERJ 33 mm, non-local (Sr)**			
Sample nr	Distance from ERJ (mm)	d13C VPDB (‰)	d18O VSMOW (‰)
MG04-01	33	-13.41	25.36
MG04-02	30.25	-13.59	25.43
MG04-03	27.5	-13.86	25.32
MG04-04	24.75	-13.64	25.82
MG04-05	22	-13.64	26.13
MG04-06	19.25	-13.64	26.62
MG04-07	16.5	-13.5	26.99
MG04-08	13.75	-13.39	27.4
MG04-09	11	-13.2	27.49
MG04-10	8.25	-12.94	28.23
MG04-11	5.5	-13.02	27.37
MG04-12	2.75	-13.03	26.96
MG04-13	0	-13.05	25.95
**MG06, Middle Iron Age B, M3i R, wear stage g, cusp to ERJ 31 mm, sample nr 10 missing**			
Sample nr	Distance from ERJ (mm)	d13C VPDB (‰)	d18O VSMOW (‰)
MG-06-01	31	-12.10	23.96
MG-06-02	28.42	-12.34	25.36
MG-06-03	25.84	-12.20	25.39
MG-06-04	23.26	-12.26	25.35
MG-06-05	20.68	-12.21	25.14
MG-06-06	18.1	-12.33	25.03
MG-06-07	15.52	-12.29	24.62
MG-06-08	12.94	-12.25	24.32
MG-06-09	10.36	-12.61	24.33
MG-06-10	7.78	-	-
MG-06-11	5.2	-12.27	23.80
MG-06-12	2.62	-12.22	23.77
MG-06-13	0	-12.17	24.96
**MG08, Middle Roman A, M3i L, wear stage g, cusp to ERJ 33 mm, sample nr 11 double length**			
Sample nr	Distance from ERJ (mm)	d13C VPDB (‰)	d18O VSMOW (‰)
MG08-01	33	-12.07	24.29
MG08-02	30	-12.05	24.39
MG08-03	27	-12.06	24.24
MG08-04	24	-12.11	24.41
MG08-05	21	-12.06	24.8
MG08-06	18	-11.99	24.98
MG08-07	15	-11.94	25.37
MG08-08	12	-11.74	25.81
MG08-09	9	-11.57	25.96
MG08-10	6	-11.64	25.59
MG08-11	0	-11.64	25.02
**MG13, Late Iron Age A, M3i L, wear stage j, cusp to ERJ 35 mm, sample nrs 1-3 wider than the others**			
Sample nr	Distance from ERJ (mm)	d13C VPDB (‰)	d18O VSMOW (‰)

MG-13-01	35	-13.76	25.96
MG-13-02	30.5	-12.21	24.77
MG-13-03	26	-11.94	24.87
MG-13-04	21.5	-11.62	24.24
MG-13-05	17.9	-11.59	24.33
MG-13-06	14.3	-11.68	24.54
MG-13-07	10.7	-11.49	24.27
MG-13-08	7.1	-11.62	24.23
MG-13-09	3.5	-11.58	24.22
MG-13-10	0	-11.88	23.98
**MG24, Middle Roman A, M3i R, wear stage g, cusp to ERJ 36 mm**			
Sample nr	Distance from ERJ (mm)	d13C VPDB (‰)	d18O VSMOW (‰)
MG-24-01	36	-12.05	24.56
MG-24-02	32.4	-12.04	23.99
MG-24-03	28.8	-12.04	23.75
MG-24-04	25.2	-11.96	23.28
MG-24-05	21.6	-12.09	23.16
MG-24-06	18	-12.06	22.87
MG-24-07	14.4	-12.00	23.01
MG-24-08	10.8	-12.00	22.93
MG-24-09	7.2	-11.89	22.82
MG-24-10	3.6	-11.88	23.13
MG-24-11	0	-11.97	23.78
**MG26, Middle Roman A, M3i R, wear stage b, cusp to ERJ 46 mm**			
Sample nr	Distance from ERJ (mm)	d13C VPDB (‰)	d18O VSMOW (‰)
MG-26-01	46	-11.95	26.84
MG-26-02	43.3	-12.10	26.53
MG-26-03	40.6	-12.16	26.13
MG-26-04	37.9	-12.22	25.51
MG-26-05	35.2	-12.19	25.40
MG-26-06	32.5	-12.11	24.75
MG-26-07	29.8	-12.08	24.49
MG-26-08	27.1	-11.93	24.39
MG-26-09	24.4	-12.08	24.54
MG-26-10	21.7	-12.08	24.24
MG-26-11	19	-11.75	24.24
MG-26-12	16.3	-12.17	24.85
MG-26-13	13.6	-12.12	25.51
MG-26-14	10.9	-11.91	25.92
MG-26-15	8.2	-11.81	26.60
MG-26-16	5.5	-11.78	26.99
MG-26-17	2.8	-11.69	26.80
MG-26-18	0	-11.65	25.86
**MG31, Middle Iron Age B, M3i r, wear stage e/f, cusp to ERJ 40 mm, non-local (Sr)**			
Sample nr	Distance from ERJ (mm)	d13C VPDB (‰)	d18O VSMOW (‰)
MG 31 -01	40	-11.62	24.57
MG 31 -02	36.4	-11.73	24.63
MG 31 -03	32.8	-11.86	24.82
MG 31 -04	29.2	-11.98	24.97
MG 31 -05	25.6	-11.94	25.39
MG 31 -06	22	-12.09	25.59
MG 31 -07	18.4	-12.24	26.35
MG 31 -08	14.8	-12.44	26.52

MG 31 -09	11.1	-12.43	26.23
MG 31 -10	7.5	-12.65	25.65
MG 31 -11	3.7	-12.84	24.78
MG 31 -12	0	-12.81	24.09
**MG33, Late Iron Age A, M3i L, wear stage g, cusp to ERJ 35 mm (missing sample nr 9 due to miscount)**			
Sample nr	Distance from ERJ (mm)	d13C VPDB (‰)	d18O VSMOW (‰)
MG-33-01	35	-11.57	24.35
MG-33-02	31.8	-11.72	23.86
MG-33-03	28.6	-11.78	23.59
MG-33-04	25.4	-11.77	23.26
MG-33-05	22.2	-11.70	22.87
MG-33-06	19	-11.71	22.97
MG-33-07	15.8	-11.80	22.72
MG-33-08	12.6	-11.90	23.40
MG-33-10	9.4	-11.66	23.79
MG-33-11	6.2	-11.48	24.27
MG-33-12	3.1	-11.36	24.72
MG-33-13	0	-11.36	24.49
**MG38, Early Roman B, M3i R, wear stage g, cusp to ERJ 40 mm**			
Sample nr	Distance from ERJ (mm)	d13C VPDB (‰)	d18O VSMOW (‰)
MG-38-1	40	-12.45	23.69
MG-38-2	36.9	-12.59	23.76
MG-38-3	33.8	-12.57	23.79
MG-38-4	30.7	-12.55	23.47
MG-38-5	27.6	-12.52	23.82
MG-38-6	24.5	-12.46	24.19
MG-38-7	21.4	-12.31	24.45
MG-38-8	18.3	-12.10	24.73
MG-38-9	15.2	-12.11	24.69
MG-38-10	12.1	-11.98	25.00
MG-38-11	9	-11.89	25.08
MG-38-12	6	-11.88	25.25
MG-38-13	2.9	-11.79	24.97
MG-38-14	0	-11.97	25.16
**MG40, Middle Iron Age b, M3i L, wear stage g, cusp to ERJ 43 mm**			
Sample nr	Distance from ERJ (mm)	d13C VPDB (‰)	d18O VSMOW (‰)
MG-40-01	43	-12.37	24.86
MG-40-02	39.93	-12.61	24.64
MG-40-03	36.86	-12.74	24.14
MG-40-04	33.79	-12.69	24.03
MG-40-05	30.72	-12.65	23.90
MG-40-06	27.65	-12.66	23.82
MG-40-07	24.58	-12.70	23.71
MG-40-08	21.51	-12.72	24.02
MG-40-09	18.44	-12.76	23.99
MG-40-10	15.37	-12.71	24.66
MG-40-11	12.3	-12.64	25.16
MG-40-12	9.23	-12.49	25.59
MG-40-13	6.16	-12.44	25.91
MG-40-14	3.09	-12.63	26.08
MG-40-15	0	-12.52	25.67
