# Supplementary material for: Cattle management in an Iron Age/Roman settlement in the Netherlands: Archaeozoological and stable isotope analysis
Source: PLoS One. 2021 Oct 1;16(10):e0258234. doi: 10.1371/journal.pone.0258234 (PMC8486104; doi:10.1371/journal.pone.0258234)
Supplement: Attachment — Submitted filename: Response to reviewers.docx [file pone.0258234.s003.docx]

**Rebuttal (author’s comments in italics)**

Academic editor’s comments:

1. Please ensure that your manuscript meets PLOS ONE's style requirements, including those for file naming. The PLOS ONE style templates can be found at 
https://journals.plos.org/plosone/s/file?id=wjVg/PLOSOne_formatting_sample_main_body.pdf and 
<https://journals.plos.org/plosone/s/file?id=ba62/PLOSOne_formatting_sample_title_authors_affiliations.pdf>

*I have checked the manucript and file names and hope they now meet the requirements.*

2. In your manuscript, please provide additional information regarding the specimens used in your study. Ensure that you have reported specimen numbers and complete repository information, including museum name and geographic location.

*I have added a table in the supplementary information with additional information on the specimens used.*

3. We note that Figure 1 in your submission contain [map/satellite] images which may be copyrighted. All PLOS content is published under the Creative Commons Attribution License (CC BY 4.0), which means that the manuscript, images, and Supporting Information files will be freely available online, and any third party is permitted to access, download, copy, distribute, and use these materials in any way, even commercially, with proper attribution. For these reasons, we cannot publish previously copyrighted maps or satellite images created using proprietary data, such as Google software (Google Maps, Street View, and Earth). For more information, see our copyright guidelines: http://journals.plos.org/plosone/s/licenses-and-copyright.
 
We require you to either (1) present written permission from the copyright holder to publish these figures specifically under the CC BY 4.0 license, or (2) remove the figures from your submission:

*Figure 1 has been replaced.*

4. Please review your reference list to ensure that it is complete and correct. If you have cited papers that have been retracted, please include the rationale for doing so in the manuscript text, or remove these references and replace them with relevant current references. Any changes to the reference list should be mentioned in the rebuttal letter that accompanies your revised manuscript. If you need to cite a retracted article, indicate the article’s retracted status in the References list and also include a citation and full reference for the retraction notice.

*I have checked my reference list and I believe it is complete. I do not think I have cited any retracted articles.*

Reviewer’s comments:

could avoid some words such as hypsodont

*I have added a brief explanation of ‘hypsodont’ the first time it is mentioned in the text.*

Page 8 Line 97: authors meaning of the previous study or this paper (or perhaps they are the same)?

*I have changed the text to “authors of the Schipluiden study” to clarify this.*

Page 10 Line 250: adding the word “either” before cheese and a comma after herb could help clarify this sentence

*done*

Page 10 Line 254: suggestion of an additional sentence summarizing the organic residue evidence for dairy—present but not ubiquitous? Not well enough studied to establish a pattern? This could be highlighted in the discussion-residue and archaeozoological remains indicate some milk exploitation, but it was not the focus? Re-establishing these findings within the new isotope findings might be impactful.

*I have added two sentences to summarize the organic residue evidence, as suggested. I have also changed the text in the discussion (“Birth season”) and conclusion somewhat to emphasise the point about organic residue. The lack of a focus on milk as shown by the archaeozoological remains already seems clear enough to me as the text is now.*

If there is space, I would like to see it come back to self-sufficiency and household-level exploitation of milk, or some ideas about why there was a shift towards decreased birthing season/prominence of milk exploitation at the end of the paper, or why this study matters more broadly. Take the conclusion one step bolder and broader.

*I have added a few sentences in the discussion on this issue.*
